# Navigating family: Immigrant‐origin Latinx youth perspectives on parental sociopolitical socialization

**DOI:** 10.1111/jora.70273

**Published:** 2026-10-07

**Authors:** Wendy de los Reyes, Victoria Vezaldenos, Corissa Draper, Rosaria Barraza

**Affiliations:** ^1^ Department of Psychological Science Claremont McKenna College Claremont California USA; ^2^ Combined Program in Education and Psychology University of Michigan Ann Arbor Michigan USA; ^3^ Department of International Affairs & Latin American Studies Florida State University Tallahassee Florida USA

**Keywords:** civic engagement, Latina/o/e/x, parenting, social justice, sociopolitical development, sociopolitical socialization, sociopolitical values

## Abstract

Latinx youth navigate unique sociopolitical contexts shaped by anti‐immigrant rhetoric and cultural values like familism. This qualitative study examined how 23 immigrant‐origin Latinx older adolescents (*M*_age_ = 20.22) perceived their parents' role in their sociopolitical development. Using a modified constructivist grounded theory, we found that parents contributed to participants' sociopolitical development through indirect (affirming Latin American identity, imparting communal values) and direct (modeling engagement, providing encouragement) socialization practices. Socialization occurred primarily through dialogue, with tensions at times emerging. These tensions sometimes were obstacles to development but also catalyzed deeper engagement. Findings extend sociopolitical development theory by documenting how family relationships function as early life experiences (i.e., socialization), showcasing the complex interplay between parents and sociopolitical development.

Sociopolitical development, the process through which individuals develop awareness, motivation, and capacity to challenge social inequalities, is critical for understanding youth engagement in social justice efforts (Watts & Halkovic, 2022). Latinx youth, particularly those who are immigrant‐origin (i.e., both U.S.‐born and foreign‐born children of immigrants), face numerous adversities within U.S. social and political systems, underscoring the importance of supporting their sociopolitical engagement (Flores‐González, 2017). The current sociopolitical context may be particularly challenging for Latinx immigrant‐origin youth as dominant rhetoric has ostracized and criminalized Latin American immigrants and their families (Ayón, 2018; Ayón et al., 2025; Blanco Martinez et al., 2023). With an increased social discourse regarding immigration processes and documentation status, Latinx families in the U.S. may be engaging in more frequent dialogue at home around these social issues. This sociopolitical context in combination with support from parents may catalyze sociopolitical development among Latinx youth—making them more aware of systemic inequities and, in turn, youth may become motivated to disrupt such systems.

## Theoretical frameworks

We situate Latinx immigrant‐origin adolescent perspectives as a unique case for the study of parental sociopolitical socialization given their ongoing development within a contentious, anti‐immigrant sociopolitical context, unique cultural values (e.g., familism), and nuanced experiences with immigration. Given this positioning, we leverage theoretical frameworks that honor the strengths of immigrant youth and situate these strengths within broader sociopolitical development theory.

### Sociopolitical development theory

Sociopolitical development is the process of working toward a more just society through challenging oppression and advancing liberation (Watts et al., 1999, 2003). Initially, scholars situated sociopolitical development within the context of Black youth's oppression and resistance in the United States (Watts et al., 1999, 2003) with theoretical foundations in liberation psychology—a field that emerged from Latin America and centers human rights and social equity (Fox & Prilleltensky, 1997; Watts & Flanagan, 2007). Watts and colleagues created sociopolitical development as a youth development framework that explicitly identifies racism as a structural obstacle to well‐being and emphasizes how Black youth can actively participate in their liberation (Hope & Jagers, 2014). This theoretical lens has been extended to understanding the experiences of a diverse group of adolescents who experience marginalization, inclusive of Latinx young people (Maker Castro et al., 2022).

The literature has also demonstrated that adolescence is a crucial developmental period for sociopolitical development. During adolescence, youth experience a myriad of physiological changes, including rapid brain development. Such brain development offers youth greater cognitive capacity to understand complex, abstract social issues especially as they operate at a systemic rather than interpersonal level (Quintana, 1998; Umaña‐Taylor et al., 2014; Watts et al., 2011; Wray‐Lake & Ballard, 2023), potentially motivating social action (Freire, 1973; Watts et al., 2011). This study centers on young people between 18 and 24 years old, at times referred to as late adolescence or emerging adulthood. During this developmental period, there is heightened change and identity exploration as they consider their life and potential pathways (e.g., worldviews, relationships, work; Arnett, 2007).

These developmental transitions position adolescents in a distinctive time to participate in sociopolitical development. In the U.S., many individuals in late adolescence finish high school and move into post‐secondary education or employment. When navigating the transition to adulthood, college settings provide an optimal context for civic engagement, as they offer increased agency and autonomy (Bloemraad & Trost, 2008; Flanagan & Levine, 2010). Further, teens at this age also start to gain greater access to civic action within the U.S. political landscape (e.g., voting, independent participation in formal organizing spaces).

Several interconnected elements comprise youth sociopolitical development: (a) social identity, (b) early upbringing (i.e., socialization), (c) critical social analysis, (d) sense of agency, (e) opportunity structures (i.e., social networks that build skills, knowledge, and abilities), and (f) sociopolitical engagement, including various forms of activism (Hope et al., 2023; Watts & Flanagan, 2007; Watts & Halkovic, 2022). A recent systematic review (Heberle et al., 2020) found that scholarship in this area has predominantly concentrated on individual‐level outcomes and has devoted less attention to contextual factors, like relationships and socialization. The current study aims to address this gap by emphasizing relational factors to expand the field's focus on contextual components in sociopolitical development. Specifically, we examine the role of parents in youth's early upbringing experiences, such as socialization from parents, which may introduce young people to awareness of social inequities and inspire engagement in addressing them.

### Risk and resilience of immigrant youth

Further, our study is informed by the *Integrative Risk and Resilience Model for the Adaptation of Immigrant‐origin Children and Youth* (Suárez‐Orozco et al., 2018), which recognizes that while Latinx youth may develop within contentious sociopolitical contexts that may harm their development (i.e., xenophobia, racism), they are also able to access support from close relationships with family which in turn positively supports their development (i.e., parents; Suárez‐Orozco et al., 2018). This framing culturally grounds our study by centering families' role in the healthy development of Latinx immigrant‐origin youth.

## Brief review of parental socialization literature

There has been emerging sociopolitical development literature examining the early upbringing experiences of racially/ethnically diverse youth, with some researchers homing in on the role of parents in particular. This scholarship highlights how parents convey both messages to their children that may encourage youth to engage in sociopolitical engagement through a process called socialization (Hope et al., 2023). A few studies examine the role of *political or civic socialization*; how parents discuss political events and model civic participation to their youth (Pacheco, 2008; White & Mistry, 2016). Among diverse marginalized youth, research has demonstrated that youth who report engaging in sociopolitical discussions at home also report greater civic agency (Bañales, Aldana, et al., 2021; Bañales, Hope, et al., 2021), commitment toward social change, and longitudinal engagement in civic behaviors (Diemer, 2012). While research on political socialization is limited, there is a larger body of literature exploring how diverse parents discuss racialized issues with their children and how such messages may relate to youth's sociopolitical engagement.

Through a process of *ethnic‐racial socialization*, parents convey both overt (i.e., explicit) and covert (i.e., subliminal) messages about their children's ethnic‐racial groups at home (Hughes et al., 2023). Parents take on a range of ethnic‐racial socialization approaches with their children ranging from those that minimize the existence of racial differences to messaging that conveys a more critical understanding of racial group membership (Vezaldenos et al., 2023). A recent systematic review has documented that Latinx parents engage in several ethnic‐racial socialization strategies with their children (Ayón et al., 2020). All but one study reviewed by Ayón et al. (2020) centered on cultural socialization, or the transmission of messages surrounding cultural values, customs, and traditions. A subset of these studies examined cultural values unique to the Latinx community like *respeto*, the importance of respecting and honoring the wishes of one's elders, and *familismo*, the centrality of the family unit in one's day‐to‐day life. Another notable, but less prevalent, strategy included preparation for bias socialization where Latinx parents aimed to warn their children about potential discrimination they may experience and impart coping strategies that they can leverage in response to such inevitable instances. Sometimes Latinx parents further promoted mistrust of other racial groups, expressing to their children the need to exercise caution around non‐Latinos as they may be perpetrators of discrimination. Interestingly, just one study identified *immigration‐related socialization* as a strategy unique to immigrant‐origin Latinx families (Ayón, 2016). With this approach parents made concerted efforts to teach their children about their home countries and explicitly discussed documentation status.

While these specific socialization practices and their links to sociopolitical development among Latinx youth have not yet been explored, racial socialization strategies broadly and their relationship to positive developmental outcomes have been examined among diverse youth. Namely, research has demonstrated that ethnic‐racial socialization can successfully foster a secure ethnic‐racial identity among diverse youth. That is, dialogue about race and racism at home can support youth in a deeper understanding of their ethnic and racial backgrounds and a positive relationship to the identities they hold (Ayón et al., 2020; Umaña‐Taylor et al., 2014). We know that youth with secure ethnic‐racial identity experience better academic, socioemotional, and mental health outcomes across the lifespan (Rivas‐Drake et al., 2014), which points to the impact parents have in supporting the positive development of their children. Furthermore, emerging scholarship has begun to identify empirical connections between parental ethnic‐racial socialization and aspects of youth sociopolitical engagement.

Research has begun to explore how ethnically, and racially diverse parents orient their children toward antiracism by aiding their youth in developing systemic understandings of social issues. In fact, a recent review of racial socialization literature documented that parent messages around race not only affirm youth's identities but also have the potential to promote civic resistance in service of antiracism (Hughes et al., 2023). Among Black adolescents quantitative research has revealed positive associations between parental racial socialization messages and components of sociopolitical development (Anyiwo et al., 2023; Bañales, Aldana, et al., 2021; Bañales, Hope, et al., 2021; Byrd & Ahn, 2020). Specifically, Bañales, Aldana, et al. (2021), Bañales, Hope, et al. (2021) found that Black teenagers who reported high levels of cultural pride socialization also reported greater critical agency. Furthermore, Anyiwo et al. (2023) identified that increased cultural socialization (e.g., conversations or experiences aimed at engaging youth in Black culture) was directly associated with higher levels of critical reflection, agency, and interpersonal, political, and online action. Similarly, high levels of racial barrier socialization (e.g., messages about potential future discrimination) was directly related to higher scores for critical reflection, critical agency, and political action. Racial barrier socialization was indirectly associated with interpersonal action by way of critical reflection and online action through critical agency respectively (Anyiwo et al., 2023).

In samples of diverse adolescents scholars have identified similar relationships between ethnic‐racial socialization and critical consciousness (Byrd & Ahn, 2020; Mejia et al., 2024). Byrd and Ahn (2020) leveraged latent profile analysis to reveal that youth with a “high discrimination” profile–that is having high racial socialization and experiences with discrimination scores–reported significantly greater levels of critical reflection, agency, and multiple forms of action. Mejia et al. (2024) found that cultural socialization and preparation for bias messages were positively related to anti‐racist action and all components of critical consciousness. Interestingly, familism, when interacted with preparation for bias messages, significantly predicted critical motivation and anti‐racist action. When familism interacted with cultural socialization, it was predictive of higher scores for critical motivation. This points to the unique potential for familism and cultural socialization messages to work in tandem to promote sociopolitical development. Given that familism is a notable protective factor within Latinx families, this merits further exploration among Latinx adolescents. Taken together, these findings suggest that various parental approaches to ethnic‐racial socialization have the potential to support sociopolitical development among adolescents in particular and a paucity of literature examining these associations within Latinx immigrant‐origin youth specifically.

Among Latinx youth, it has been found that cultural and sociopolitical discussions with parents were positively related to feeling greater collective responsibility and expectations for future community involvement (Pinetta et al., 2020). Additional research has shown that cultural socialization from Latinx parents is directly related to youth caring for their communities (Blanco Martinez et al., 2022). This study also found an indirect association between cultural socialization and youth social responsibility by way of Latinx parent sociopolitical discussions and civic socialization (Blanco Martinez et al., 2022). Qualitative work appears to support this general trend as Blanco Martinez et al. (2023) identified that parents reported engaging in both sociopolitical and ethnic‐racial socialization at home, particularly as it pertained to documentation status. Their youth felt that these conversations were essential, particularly within challenging sociopolitical contexts, and that such discussions helped them to develop an awareness of oppression and motivation to take action against it. These findings demonstrate that Latinx parents' discussions of culture and politics at home may support youth in their sociopolitical development. However, these phenomena merit further exploration qualitatively. Additionally, it is unclear whether or how parental messages might *hinder* youth sociopolitical development.

## The unique positionality of Latinx immigrant families

Latinx immigrant families offer a unique social context for the study of sociopolitical socialization among older adolescents due to their positionalities as immigrants and their unique cultural values (e.g., familism) particularly within the current sociopolitical context. Familism refers to a deep bond with family members and a related set of beliefs that prioritizes allegiance to family, receiving support from family, and feeling a duty to honor one's family (Steidel & Contreras, 2003). Familism is commonly studied within Latinx families due to Latin American cultural emphasis on family values, loyalty, and identity (Gibbons, 2024). However, links between familism and youth sociopolitical development remain largely unexplored. Familism as a cultural value may uniquely position Latinx parents for sociopolitical socialization. Because familism centers allegiance and loyalty to family, the perspectives of parents may be particularly salient for Latinx immigrant‐origin youth. With pressures to make their family proud and to maintain a strong family identity, parents of Latinx youth might have the unique ability to both promote and inhibit their child's sociopolitical development competencies.

Furthermore, immigrant‐origin Latinx youth have proximal experiences with immigration either through their own experiences or by way of their parents. These youth are acculturating to the U.S. context in a historical period that actively criminalizes Latin American immigrants and their families. This may be particularly stressful for Latinx youth from mixed‐status families—meaning youth and their family members may have different documentation statuses they are navigating (undocumented, DACAmented, green card holders etc.; Ayón, 2018; Blanco Martinez et al., 2023; Sánchez et al., 2022). Therefore, social issues like those pertaining to immigration and deportation may be particularly relevant to Latinx immigrant‐origin adolescents and their families which may manifest as daily conversations regarding social issues at home, thus prompting sociopolitical development among youth. Furthermore, the context from which the family emigrated may pose relevance for SPD. For instance, if families were fleeing political violence or instability from their birth countries, they may already be aware of sociopolitical issues from an international perspective which may prompt sociopolitical development among youth growing up in the U.S. These distinguishing characteristics position Latinx‐immigrant origin families as a unique context for the study of sociopolitical development. By exploring youth perceptions of their parents' sociopolitical socialization, researchers will have a lens to understand how cultural assets and positionality operate within contentious sociopolitical contexts to produce socially conscious youth that are motivated to engage in sociopolitical action.

## CURRENT STUDY

Existing literature demonstrates the benefits of sociopolitical engagement among diverse youth. While literature has begun to explore how non‐familial adults orient youth toward social change, research is limited on how parents might support youth sociopolitical development. Scholarship on parental socialization practices within Latinx families primarily centers on ethnic‐racial socialization and how broader messages about culture can inform sociopolitical engagement and beliefs among youth. There is limited research on the explicit strategies Latinx parents take to encourage sociopolitical development among their children, and no studies, to our knowledge, examine this qualitatively from the youth's perspective. In an effort to address these intersecting gaps in the literature, we leverage Constructivist Grounded Theory (Charmaz, 2014) to explore: *How do immigrant‐origin Latinx youth perceive the role of their parents in their sociopolitical development?*

## METHOD

We used a modified constructivist grounded theory approach (Charmaz, 2014) to develop a theory of how immigrant‐origin Latinx adolescents perceive the role of their parents in their sociopolitical development. This approach uses a social constructivist epistemology and assumes that the researcher is part of the world being studied and the data collected. Thus, the researcher offers an interpretation of the data instead of an exact picture (Andrews, 2012; Charmaz, 2014). This approach was particularly well‐suited for our research aims, as it allowed us to remain open to emergent categories while being attentive to the specific cultural contexts and family dynamics that characterize Latinx immigrant families. In light of this approach, we will begin with a reflection of the research team's positioning within this study.

### Researcher positionality

Throughout the development of this study, the authors reflected on how their own backgrounds and identities informed the lens with which they approached the work. The first author, WDLR, led the development of this study and conducted all interviews. She started each interview by briefly describing her background and interest in the project to ease participants' comfort level. She identifies as a Cuban‐born Latina who migrated to the U.S. at age six and grew up in a predominately Latinx community. She is also the parent of two young children. The second author, VV, identifies as a Biracial Mexican‐White granddaughter of immigrants and was raised in a predominantly Latinx, agricultural community in California; the third author, CD, identifies as a White, cisgender woman raised in a predominantly white town in the U.S.; and the fourth author, RB, identifies as a Peruvian‐born Latina who migrated to the U.S at the age of seven and grew up in a predominately Latinx urban area. CD and RB were 19‐year‐olds at the start of data analysis, within the same age range as study participants. As a research team, our intersecting identities offer us variable insight into the immigrant experience–through our own migration experiences and/or engagement in activism–across several U.S. states in rural, urban, and suburban areas. Furthermore, our individual engagement in social justice work has allowed us to recognize both opportunities and barriers to sociopolitical engagement for youth.

Our ongoing, personal journeys toward antiracism inform our interests in promoting opportunity structures that aim to engage youth in this work. As a result, we approach the current study with a strengths‐based lens recognizing that while Latinx youth may encounter several obstacles within their sociopolitical development, there are also opportunity structures and social supports that play pivotal roles in supporting their ongoing engagement. Despite the proximal and distal challenges youth face, we as a research team believe that youth have the tools necessary to recognize, challenge, and reform social systems that continue to reproduce harm on Latinx communities.

### Youth collaborators and organizational partners

To involve young people throughout various stages of the research, we established a youth research advisory council. This approach reflected a commitment to youth engagement along a continuum that offered young people decision‐making authority in select aspects of the research—a strategy designed to mitigate potential constraints within our research team and to provide richer context for understanding participants' lived experiences (Hart, 1992). About a year before data collection began, WDLR partnered with a Florida high school student in her social network to assemble a group of six Latinx immigrant‐origin adolescents ranging from 16 to 18 years old (*M* = 17.17; 50% male; 50% female; 1 in college and the rest in high school), representing diverse national origins including Peru, Nicaragua, Argentina, Cuba, Honduras, and Mexico. This advisory council collaborated in developing the initial draft of the interview protocol and provided feedback on subsequent drafts with a focus on the accessibility/readability of questions in a final interview protocol. As the project lead, WDLR was in her early 30s when data collection took place; her primary concern was to ensure that the language of the interview questions was accessible to the participants, especially the youngest participants who were just finishing high school and entering college. Among these youth was RB, who subsequently joined the broader project as a paid research assistant and coauthor of this paper, drawing on her substantial community organizing background. All youth advisory council members received compensation in the form of gift cards, honoraria, or stipends as recognition of their contributions.

Our additional partnerships included collaborators from academic institutions and non‐profit organizations. We worked with two large commuter universities in major urban centers: a prominent public university in Florida recognized as a Hispanic‐Serving Institution, and a private, mid‐sized Catholic university in Illinois serving a predominantly White student population. Additionally, we partnered with a national youth mentoring organization. We worked with their partners in California, Florida, New York, and Texas to strategically recruit participants from states with large and varying Latinx populations.

### Participants

The 23 participants all self‐identified as Hispanic, Latina/o, or Latinx, with ages spanning from 18 to 24 years old (*M*_age_ = 20.22). Table 1 provides detailed demographic information for each participant. The sample was predominantly female (*n* = 18) and represented 12 Latin American countries and was characterized by their diverse racial and sexual identities. When describing their family's socioeconomic background, most participants characterized it as “low‐income.” Over half the participants (*n* = 11) did not have a parent who graduated high school, and only five participants had a parent with a college degree. Thus, parental employment was typically in low‐wage positions commonly held by immigrants (e.g., construction worker, landscaping, housekeeper).

**TABLE 1:** Demographic characteristics of participants.

Participant	Age	Gender	Racial identity^a^	Sexual orientation	Educational attainment	Grew up	Current location	Gen status	Parents' country of origin	Parents' educational attainment and employment
Alejandra	19	W	Mexican	Heterosexual	In college (2nd year)	TX	TX	1st (3)	Mexico	*M*: <HS, seamstress *SF*: HS grad, Highway roads inspector
Allison	24	W	Multiracial	Heterosexual	In a master's program	CA	CA	2nd	Mexico	*M:* HS grad, Dental receptionist *F*: <HS, Construction
Andrea	19	W	Indigenous, White	Heterosexual	In college (2nd year)	FL	FL	1st (<1)	Puerto Rico	*M*: Technical degree, Preschool teacher *F:* HS grad, A/C tech
Camila	19	W	Black	Bisexual	In college (1st year)	FL	FL	2nd	Brazil, Honduras	*M:* <HS, cleaning *F:* <HS, Landscaping
Catalina	23	W	Indigenous, White	Heterosexual	Bachelor's degree	MD and TX	DC	1st (5)	Colombia, Ecuador	*M:* Bachelor's degree, IT help *F:* Bachelor's degree, Data center employee
Carla	20	W	Multiracial	Bisexual	In college (2nd year)	FL	FL	1st (3)	Dominican Republic	*M*: <HS, self‐employed *F*: N/A
Cris	18	GF	Indigenous, White	Bisexual	In college (1st year)	VA	FL	2nd	Honduras	*M:* Some HS but no degree, NA *F*: <HS, Restaurant industry
Fatima	20	W	Brown	Queer	In college (2nd year)	FL	FL	2nd	Colombia, Cuba	*M*: Bachelor's degree^b^, Bookkeeper *F*: <HS, Deceased
Fernanda	20	W	Indigenous	Bisexual	In college (3rd year)	NY	NY	2nd	Mexico	*M:* <HS, Housewife *F:* Some HS but no degree, Laborer
Gabriela	18	W	Multiracial	Bisexual	In college (1st year)	CO	MA	2nd	El Salvador	*M:* <HS, N/A *F:* <HS, Landscaping
Luciana	18	W	White	Heterosexual	In college (1st year)	IL	IL	2nd	Mexico	*M:* HS grad, N/A *F:* Technical degree, Mechanic
Matias	18	M	Indigenous; White	Heterosexual	In college (1st year)	FL	FL	2nd	Cuba, Honduras	*M:* <HS, Housekeeper *F:* <HS, Chauffer
Mia	20	W	Multiracial, Asian, Indigenous, White	Bicurious	In college (3rd year)	IL	IL	2.5	Peru	*M:* Bachelor's degree, Family advocate for a domestic violence agency *F*: Bachelor's degree, Works two jobs in healthcare
Nicolas	21	M	Multiracial, Latino	Queer	In college (3rd year)	TX	VA	2nd	Mexico	*M*: <HS. Office Secretary *F:* HS grad, Manager
Olivia	21	W	Indigenous, White	Asexual	In college (4th year)	VA	VA	2nd	Mexico	*M:* <HS. Stay‐at‐home mom *F:* <HS, Contractor
Samuel	19	M	White	Heterosexual	In college (1st year)	TX	TX	2nd	Mexico	*M*: Some HS but did not graduate, Cashier *F:* <HS, Construction
Santiago	22	M	Hispanic	Heterosexual	In college (transfer)	Lima, Peru	NY	1st (17)	Peru	*M:* HS grad, housekeeper. *F:* HS grad, N/A (In Peru)
Sara	23	W	White	Bisexual	Bachelor's degree	FL	FL	2nd	Colombia	*M:* HS grad. Self‐employed beauty salesperson *F:* HS grad, Stonemason
Sofia	21	W	Multiracial, White	Heterosexual	In college (3rd year)	FL	FL	2.5	Guatemala	*M*: Graduate degree, Licensed clinical social worker and psychotherapist *F:* HS grad, Disability
Valentina	18	W	Indigenous	Asexual	In college (1st year)	FL	FL	2nd	Colombia, Nicaragua	*M:* Graduate degree, Nurse *F:* Technical degree, Construction manager
Vanessa	19	W	White	Heterosexual	In college (1st year)	IL	IL	2nd	Mexico	*M:* <HS, Nail technician *F:* <HS, NA
Victoria	21	W	Hispanic	Heterosexual	In college (4th year)	IL	IL	2nd	Mexico	M: <HS, Stay‐at‐home mom *F:* <HS. Chef
Ximena	24	W	Indigenous	Heterosexual	Master's degree	FL	VA	1st (1)	Mexico	*M:* Bachelor's degree, housekeeper *F:* Bachelor's degree, business owner

Abbreviations: GF, gender fluid; M, man; W, woman.

^a^ Self‐reported racial identity along with identifying as Hispanic or Latin/a/o/x/e. Educational Attainment: Current status (Year in school if in undergrad). Gen Status = Generational Status: 1st = Born outside of the U.S. (age of arrival in years in parenthesis), 2nd = Both parents were born outside the U.S, 2.5 = one parent was born outside of the U.S., M = Mother, F = Father, SF = Stepfather. N/A = no answer or unknown.

^b^ Foreign degree did apply in the U.S.

Study eligibility criteria required that participants: (a) identified as Hispanic, Latina/o, or Latinx, (b) had at least one parent born outside the United States, (c) were 18‐ to 24‐years‐old, (d) were English or Spanish speakers, and (e) expressed interest in contributing to community change. Recruitment occurred through multiple channels. Partners from the aforementioned national mentoring organization reached out to youth participating in local mentoring programs across California, Florida, New York, and Texas. Supplementary recruitment efforts included introductory course announcements, email outreach, and distributing physical flyers; in addition to in‐person announcements at Latinx‐focused student organizations and resource centers on the two aforementioned partnering university campuses. A small number of participants also joined through the first author's personal and professional networks. Informed by our sampling strategy, maximum variation, we used a screening questionnaire to select interview participants of diverse demographic profiles (including age, gender, and country of origin) and a wide range of experiences in sociopolitical action. For example, toward the end of our data collection, we primarily focused on recruiting Latinx men as well as participants with high levels of action to fill gaps that we had been missing. As a result, we excluded 6 cisgender women with little to no levels of action.

### Procedures

This study was approved by the DePaul University institutional review board (#IRB‐2022‐596). Prospective participants initially completed an online screening survey that provided information to inform participant selection decisions. Those selected for participation completed individual Zoom interviews conducted between October 2022 and June 2023, with interview durations spanning 50–90 min (*M* = 74 min). We audio recorded all interviews, transcribed verbatim using Rev. (a transcription application), and then checked for potential mistakes. While interviews were conducted predominantly in English, Spanish phrases came up throughout some conversations. Each participant received a $20 gift card as compensation for their interview participation.

### Interview guide

With the support of our Latinx youth research advisory council, the research team members, and our collaborator from the mentoring organization, we developed a semi‐structured interview guide informed by previous studies on political engagement and sociopolitical development (Dalton, 2020; Monjaras‐Gaytán et al., 2021). We also conducted three pilot interviews prior to the start of data collection and adjusted the questions and sequence accordingly. The goal of the larger study was to ask participants to reflect on social issues important to them, action they have taken to address those issues, and how people in their lives have supported or hindered their engagement. A portion of the questions focused on how participants' parents supported and hindered their exploration of social issues and action throughout their adolescence (i.e., the focus of the current study).

To inquire how their parents influenced their involvement in social issues, we asked: “How have your parents' or guardians' views influenced your views?” Depending on their responses, we followed up with additional questions to get a better understanding of their relationship dynamics around social issues: “Do they support your views? How or why not? Do you and your parents agree on certain social issues and what should be done? Do either of your parents do any kind of activism? How have your parents' experiences in your native countries influenced your beliefs?”

### Data analysis approach

Data were analyzed using a modified Constructivist Grounded Theory approach (Charmaz, 2014). Using Dedoose, a qualitative analysis software (version 10.0.59), the first, third, and fourth authors analyzed the data in three steps: open coding, focused coding, and theoretical coding. We developed open codes during an iterative data collection and analysis process. During open coding, data were coded through an inductive approach, allowing for initial conceptualization of ideas based on our research questions, while also remaining open to any new ideas that became apparent during analysis. Due to logistical barriers in timeline, we modified our constructivist grounded theory approach through entering the focused coding phase after data collection was completed (i.e., identifying and finalizing the set of most relevant codes to categorize the data). In this phase, the first, third, and fourth authors collaboratively reviewed our preliminary open codes, refined code names, definitions, and our preliminary coding framework. The third and fourth authors then systematically re‐coded all 23 transcripts to verify that our codes remained faithful to the updated definitions while assessing for potential categories across the dataset. After focused coding, we conducted theoretical coding (i.e., conceptual coding, selective coding), in which we identified how the categories were related to each other through diagramming the central category and its major processes (Saldaña, 2021) as they relate to the research questions. The theoretical coding process involved all four authors. Upon any disagreements throughout this process, we discussed our differing points of view until we reached consensus. We wrote detailed memos throughout the coding process and kept record of our evolving coding framework to document decisions at each step.

We used four strategies to establish the trustworthiness of our analyses, attending to credibility, transferability, dependability, and confirmability (Shenton, 2004). *Credibility* (i.e., whether the findings reflect participants' realities) can be partially established by close familiarity with the culture of participants. Three of the four co‐authors are members of Latinx communities, which informed our interpretation of participants' experiences. Latinx communities are vastly diverse across the U.S., but we intentionally assembled a research team with diverse Latinx backgrounds and ages so that our multiple viewpoints could shape the analysis. The coders also engaged in negative case analysis (Lincoln & Guba, 1986), in which we actively looked for examples within the data that contradicted the findings to think critically about the results and then make any needed revisions. We chronicled this process in our memos. We also conducted steps to enhance *transferability*, which refers to a description of the study context that allows the reader to decide whether the context of this study can apply to future contexts. This included generating a detailed description of the participants (e.g., age, generational status), their contexts, and the period in which data were collected to clearly contextualize our findings for the reader. We established *dependability*, which refers to the ability to repeat the study, through a detailed description of the methods and the subsequent coding steps and decisions. We established *confirmability*, which is the process of demonstrating the steps taken to ensure findings truly emerged from the data, by recruiting a research team of differing ages and Latinx nationalities so that multiple perspectives informed the analysis and interpretation of findings. The team also reflected on their beliefs and assumptions about the data and participants. For example, the four of us discussed our understanding of power dynamics within the Latinx community, especially as it referred to family. Several of us also simultaneously experienced current events that the participants discussed, like how youth organized the school walkouts after the Parkland School Shooting. The third and fourth authors were both high school students where walkouts took place and the first author had many conversations with younger family members who also participated in these protests. Furthermore, we remain transparent in our discussed methodological limitations and our limitations as researchers, which were mentioned in the positionality section.

## RESULTS

Our findings demonstrate the nuanced ways Latinx adolescents perceived their parents' role in their own sociopolitical development, which we refer to as sociopolitical socialization. Youth in our study perceived their parents as socializing them toward their sociopolitical values, indirectly and directly throughout adolescence. Indirect socialization involved actions or conversations that were less clearly connected to the participants' sociopolitical development. Parents demonstrated indirect socialization through affirming their Latin American identity and teaching communal values. Direct socialization involved actions or conversations that had a clear connection to the participants' sociopolitical development (e.g., a parent taking their child with them to a protest). This occurred through parents modeling sociopolitical engagement and providing support for participants' own exploration of social issues.

Parental socialization was largely dialogue‐focused; parents taught communal values often through conversation, participants explored social issues by discussing them with parents, and disagreements (i.e., tensions) around social issues or values brought about arguments. These tensions with their parents were obstacles for participants' sociopolitical development, hindering their ability to engage in dialogue or action. Parental tensions were also occasionally motivating for participants, and in some cases, a catalyst for their sociopolitical development. See Figure 1 for a visual display conveying the relations between these key categories. See Table 2 for a summary of study categories, with definitions and exemplary quotes.

**FIGURE 1:**
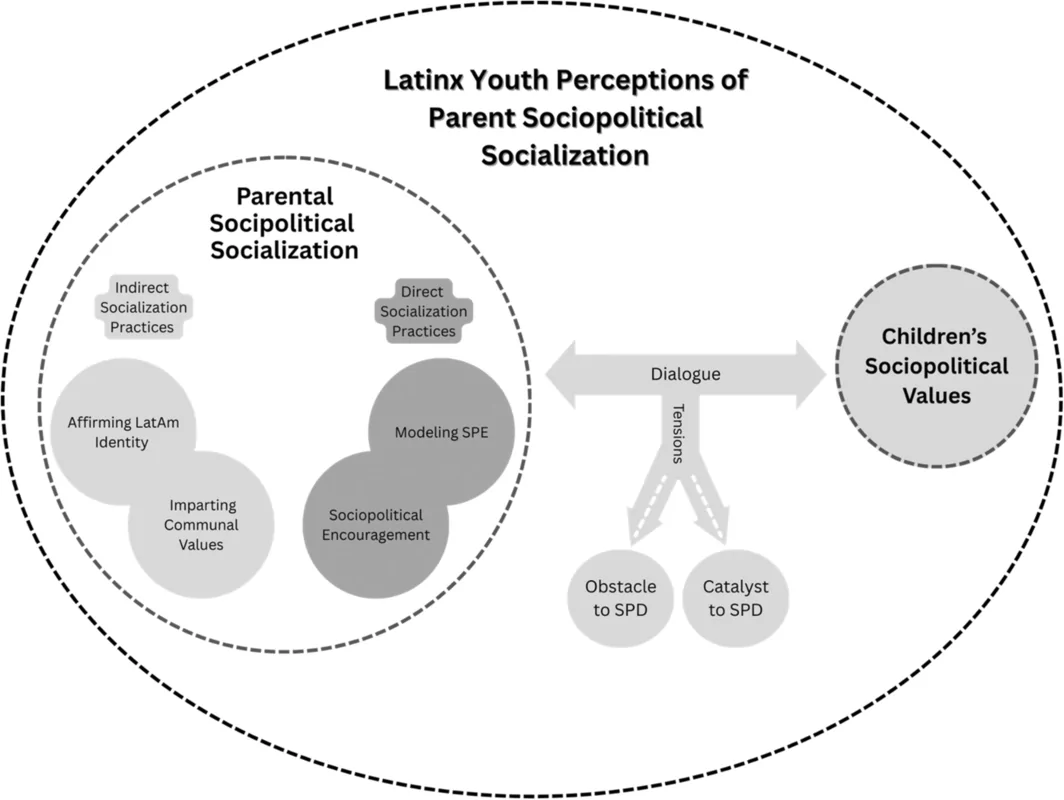
Relations between key categories. LatAm, Latin American; SPD, sociopolitical development; SPE, sociopolitical engagement.

**TABLE 2:** Summary of study categories.

Category	Definition	Exemplary quote
Affirming Latin‐American Identity	Participants discussed how their parents understood and affirmed aspects of their Latin American identity (e.g., ethnicity, nationality, race) and how these interactions had a positive impact both on their relationship to one another and their later understandings of sociopolitical issues	“With my mom, a lot of things that we talk about or think about are immigration. It never really felt like we were talking politics or we were talking in a way of teaching or a discussion. It always just felt like telling people's stories. It just felt like that's just a human experience.” (Sofia)
Imparting Communal Values	The discussion of socialization messages from their parents that indirectly impacted their sociopolitical values, participants reported that parents emphasized the importance of community. This was done through conversation and by witnessing the parents active role in the community. It provided an entry point for participants' understanding of systemic inequities	“We live in such a world that's very lacking in empathy and [my mom] always taught me that if I can help, I should always help. If that's with my time, my voice, whatever, I always need to help. She's definitely always instilled that importance in me and also the reward that it brings when you help others” (Carla)
Modeling Sociopolitical Engagement	Participants described observing and sometimes accompanying their parents in various forms of sociopolitical engagement while they were growing up. Religion played a large role in parents modeling engagement, with participants often stating that they were the first introduced to involvement in social issues through community initiatives set up by their place of worship, which parents also engaged	“Growing up, I took ballet and [my parents] made sure that when our dance studio was having drives, we would participate and make goodie bags. My mom was always the first one to volunteer to help with anything because my school was very Hispanic populated. She would always help and take kids to school if they couldn't. I grew up living in a trailer park, so there was a lot of poverty. She always made sure that kids were dressed and always felt bad. She would buy kids jackets and book bags if she saw they didn't have it” (Olivia)
Sociopolitical Encouragement	Participants described how their parents provided emotional and validation support for both their beliefs around and engagement with social issues	“My mom has always been like a person that's like, ‘You believe in what you believe in, always stick to it.’ So, I've always grown up with a very strong inner personality ‘cuz I'm very nice outside and sensitive. But inside, my core is very strong. So, it didn't really matter to me. I could be friends with someone that believes in something else because I do have family that does believe in something that I have” (Valentina)
Tension as an Obstacle to Youth Sociopolitical Development	Participants described several ways that tensions with their parents hindered their sociopolitical development. When participants felt unsupported by parents, they typically didn't want to have conversations with them about social issues. These obstacles manifested in multiple forms, including parents discouraging dialogue about social issues within the home, as well as action they wanted to take	“I definitely argue with my dad a lot on the line. We talked a lot about politics and we are not on the same page at all. Because, you know, a lot of Cubans are *very* conservative and half of my family is Cuban. Like, the people who live here are Cuban. So, I don't really have conversations with my family, and with him, not at all” (Fatima)
Tension as a Catalyst for Sociopolitical Development	For some participants, tensions with parents were instead a catalyst for their sociopolitical development	“I guess they kind of dis‐influenced me to do some stuff, but at the switch of it, kind of motivates me to be like, no, like I'm gonna keep fighting for what I believe is right. And hope that my children and their children live way better than what my own parents went through, you know?” (Allison)

### Indirect sociopolitical socialization practices

Participants described their parents as indirectly shaping their development of sociopolitical values in two keyways: affirming their Latin American identity and imparting communal values. These messages provided participants with foundational frameworks that indirectly shaped their understandings of social issues and structural inequalities. These indirect practices manifested from everyday family interactions. While these daily conversations may not have explicitly touched on social or political issues, from the perspectives of our participants, messages about their Latin American identity and community laid the groundwork for the development of their sociopolitical values.

#### Affirming Latin American identity

Participants discussed how their parents understood and affirmed aspects of their Latin American identity (e.g., ethnicity, nationality, race) and how these interactions had a positive impact both on their relationship to one another and their later understandings of sociopolitical issues. Mia, a Multiracial (20, second‐generation Peruvian), discussed how her Peruvian dad always prioritized instilling pride in her Peruvian culture through subliminal exposure to cultural customs like cooking and eating Peruvian food at home. Through sharing identities with Mia, he emphasized eating Peruvian food as a means for connecting with her culture. Her White (non‐Latina) mother chose to engage in more direct conversations about her Multiracial background. Mia recalls her mom explicitly emphasizing the importance of understanding and connecting with her multiple heritages. In recalling these moments with her parents, Mia states, “I really appreciate that and I think that that's made it easier for me to hold onto my culture being someone that's mixed.” She goes on to explain that because both of her parents emphasized connection to her Latin American culture, she later felt more passionate about taking action to address social issues that impact Latin American communities such as immigration and racism. Thus, her parents' affirmation of her Peruvian identity ultimately shaped her own sociopolitical values, even when political issues were not explicitly discussed at home.

Ximena (24, first‐generation Mexican) also discussed how because she and her parents have shared experiences with immigrating, she felt like they had a shared understanding of the challenges that came with their migration to the U.S. from Latin America. Ximena describes that having shared identity as immigrants served as an entry point for connection and positive conversation with her parents about identity‐based issues. This identity affirming space allowed her family to discuss immigration issues in a candid way, often centering their personal experiences with migration as a family. In her interview, Ximena describes that this bond with her parents around their shared immigrant identity prompted her later exploration of immigration issues. Because she felt affirmed in her identity as a Latin American immigrant at home, Ximena leveraged this supportive environment to later explore social issues that were personally relevant to her, thus prompting the formation of her own sociopolitical values.

#### Imparting communal values

When discussing socialization messages from their parents that indirectly impacted their sociopolitical values, participants reported that parents emphasized the importance of community. This was done through conversation and by witnessing the parents' active role in the community (e.g., through the church). These occurrences, while not often explicitly related to systemic issues, provided an entry point for our participants' understandings of systemic inequities. For example, participants described these early experiences as signaling to them that some people need community support. These messages imparted a basic understanding of resource disparities, which participants described as a necessary starting point which positioned them to further explore and engage with more complex social issues in their adolescence.

Sofia (21, second‐generation Guatemalan) discussed how her mom would teach her and her siblings to demonstrate more empathy toward others at school as a child. In her interview, she describes that these general life lessons served as a necessary foundation, informing her ongoing engagement with social issues. Additionally, Valentina (18, second‐generation Colombian‐Nicaraguan) shared a similar experience, saying:

[Unhoused people] would have all their stuff in shopping carts. So it was really sad and my mom would always give spare change to them…after I saw a lot of that, every time when I was in school when someone wouldn't have lunch, I would give them my lunch…like half of my lunch or like a snack that I would have… I felt like I was more aware that some people were struggling even at a young age. And I would always try to help…I feel like a lot of that came from my childhood and my mom bringing awareness to me that there are different life structures, there are different people that live through different things and that's why you have to be caring.

Valentina's mom modeled how to provide interpersonal community support—by giving spare change to unhoused people—which spurred Valentina to provide support for her peers in need. While these individual acts of kindness were not explicitly connected to systems of oppression, Valentina mentioned that this modeling from her mother made her aware of various “life structures,” alluding to a budding perspective on systemic inequality.

Catalina (23, first‐generation Colombian‐Ecuadorian) mentioned that her parents didn't discuss politics with her growing up. However, the communal values that they imparted helped shape her sociopolitical values, saying, “I think that they always set me up to be like an accepting person. They taught me to be a good human. And maybe that's how they set up my politics. But I would say that I'm actually very grateful to them because they kind of let me figure it out all by myself.” The values Catalina's parents established centered on recognizing shared humanity across all people and acceptance of difference. These perspectives are now at the core of Catalina's own sociopolitical values. She conveys that the hands‐off approach her parents took regarding politics was a good thing; as the distance allowed her to develop her own ideas about social justice in a non‐judgmental, non‐pressuring environment.

### Direct sociopolitical socialization practices

In addition to the familial conversations that indirectly shaped participants' own sociopolitical values, adolescents we spoke with also identified explicit ways their parents assisted their sociopolitical development. These experiences operated on two levels: modeling sociopolitical engagement and sociopolitical encouragement. Notably, this support transcended ideological agreement for some—parents provided encouragement and created space for their children's exploration of social issues even when they held different political beliefs. The following examples illustrate how forms of parental support that are more directly related to social issues, such as modeling sociopolitical engagement and sociopolitical encouragement, shaped participants' own confidence and sustained their commitment to social issues.

#### Modeling sociopolitical engagement

Participants described observing and sometimes accompanying their parents in various forms of sociopolitical engagement while they were growing up. Religion played a large role in parents modeling engagement, with participants often stating that they were first introduced to involvement in social issues through community initiatives set up by their places of worship, in which parents also engaged. Participants described how their parents role‐modeled giving back through church food drives, and how they often joined parents in those events. Luciana (18, second‐generation Mexican) said that, “if my parents were gonna go to a walkout, or to volunteer, talk to an alderman, [etc.], I would be there too.” As a child, Luciana's parents always had her accompany them when they engaged in social justice work. While she admits she was not an active participant at the time, Luciana goes on to describe that by being exposed to this context in her youth, she felt comfortable engaging in activism that aligned with her own sociopolitical values later in her adolescence.

At times, modeling sociopolitical engagement resembled forms of community care. Sara (23, second‐generation Colombian) recognized her mom's engagement with the community as a child and reported feeling inspired by her mother's commitment. Sara reflected on how her mother inspired her, saying:

My mom actually is a little community organizer, which I didn't realize until recently. She just finds these networks with the other Hispanic women in her area and gives them transportation to doctor's appointments or provides social support if they are undocumented and in the process of seeking citizenship, and then providing resources. She's constantly switching furniture and clothes back and forth between people in different countries. So just seeing grassroots organizing kind of in its most pure form where she doesn't get any recognition for it; she doesn't even know that that's what she's doing, but she's just naturally been doing it her whole life. That's kind of cool and it's something I really didn't even realize until recently when I've come into my identity as a grassroots organizer.

As Sara watched her mom organize and support their community. She learned the importance of giving back. Her mother's engagement was a core component of her childhood, but interestingly, she didn't recognize her mother's efforts as sociopolitical activism until she herself began engaging in grassroots efforts. This example demonstrates how parents have the ability to model sociopolitical behaviors for their children and that these models can spark future engagement in sociopolitical issues.

Participants in our sample further described how their parents took part in the sociopolitical context of their nations of origin, and thus modeling transnational sociopolitical engagement to their children. Carla (20, first‐generation Dominican) saw her mom taking such action both locally in the U.S. and in the Dominican Republic, which she described as her mom:

Go[ing] to prisons and talk[ing] to the inmates, as a friend. I remember she would host different events for the homeless, provid[ing] them with food and different necessities like clothes. So yeah, my mom is an activist locally in the Dominican Republic with the way that she can help. And even here, my mom has done different food drives, especially in 2020, helping our neighbors if they were struggling…my mom has definitely been her own activist at hand. She's just very empathetic, I feel like I inherited that from her. Any way that she can help someone she knows, a neighbor, she always does it. Recently she's helping fund a school in the Dominican Republic for homeless children and orphan children, as well. She's working with someone there to be able to like, provide these kids with education and shelter.

Carla's mother's engagement in both the United States and in the Dominican Republic motivated her to approach engagement with the same empathy that her mom demonstrated across both countries. This showed Carla the importance of engaging with social justice work as it pertains to multiple systemic issues, namely the prison system, housing, food insecurity, and schooling. Carla, now as a college student, is actively involved with student organizations that advocate for a range of social issues, such as environmental justice and LGBTQ+ rights.

#### Sociopolitical encouragement

Participants described how their parents provided emotional and validation support for both their beliefs around and engagement with social issues. This support looked like: parents encouraging their children to stand firm in their convictions, actively listening to their children's perspectives on social issues, expressing belief in their child's sociopolitical capabilities, and at times joining child‐initiated engagement. Notably, this empathetic perspective was particularly meaningful when parents held conflicting political views, with participants emphasizing the importance of being heard and having their concerns taken seriously by family members despite differences in beliefs.

Carla described how she received support from her parents around her activism. This care was especially helpful for her sociopolitical development when she felt that support was lacking from others in her community or when facing institutional pushback for her engagement from her teachers in her conservative Christian high school. She reflected on her mother's compassion, saying:

I'm super appreciative of my mom because I know a lot of people's parents aren't like that or they don't listen to them about certain things. So, it's very nice knowing that I have someone that I can talk to and someone that believes me and cares for me.

In this excerpt, Carla highlights the importance of youth feeling heard by their parents when sharing sociopolitical concerns–something she feels many youth do not receive. In her interview, she went on to describe how her mom offered advice regarding her ongoing engagement with racial justice issues. Specifically, Carla's mother encouraged her to prioritize self‐care by taking breaks from contentious political environments that she believed were causing her daughter to burn out, especially during the COVID‐19 pandemic. Carla also mentioned that her mom offered this support even when their views on current social issues differed, “I tell her my side and I know that she processes and listens to it, it brings me peace. I know that, even if it might not be immediate, it might change something within her; that makes me really happy.” Even when differing perspectives were present, Carla felt safe enough to share her perspectives with her mom, knowing that she would be listened to and emotionally supported regardless of their differences. This supportive environment encouraged further discussion of social issues at home which in turn allowed Carla to develop her own sociopolitical values.

Occasionally, participants described that their parents demonstrated further support for their engagement in sociopolitical issues by joining them in civic sociopolitical activities such as volunteering or participating in food drives. These instances were particularly impactful for our participants as parents not only listened to their child's concerns but were interested in joining their child's sociopolitical efforts when invited. In one example of this, Sara detailed how her family supported her environmental initiatives, saying:

In high school, I was able to convince them to stop buying milk and start buying almond milk. So even in little ways like that, having them hear what I had to say about the environment or issues that I had.

Her family tangibly supported her sociopolitical work in the ways that they could, like joining her fight for environmental justice by switching to an alternative milk. While it may seem like a small adjustment, her parents' actions made Sara feel supported, specifically because it demonstrated that her parents not only listened to her passions but also wanted to join her efforts for environmental justice.

### Tensions between parents and adolescents

As the previous examples demonstrate, much of participants' parental sociopolitical socialization was facilitated by dialogue. While the socialization practices described thus far demonstrate how parental messages both directly and indirectly supported youth's own development of sociopolitical values, we also found instances of tension between parent and children. As exploration occurred, participants described coming to develop their own distinct sociopolitical values and ideas of social justice, which sometimes clashed with their parents' values. These tensions stemmed from conversations about social issues divulging into arguments, where participants described being unable to see eye to eye with their parents due to conflicting sociopolitical values. These tensions sometimes became obstacles for youth sociopolitical engagement, and other times it was a catalyst for greater engagement.

#### Tension as an obstacle to youth sociopolitical development

Participants described several ways that tensions with their parents hindered their sociopolitical development. When participants felt unsupported by parents, they typically didn't want to have conversations with them about social issues. These obstacles manifested in multiple forms, including parents discouraging dialogue about social issues within the home, as well as actions they wanted to take. For example, Alejandra (19, first‐generation Mexican) discussed how her mom discouraged her engagement, believing it was unnecessary and that people who are struggling should toughen up. Alejandra felt her mom lacked sympathy for those she advocated for simply because her mother had gone through similar experiences without support. Similarly, Valentina discussed how she felt discouraged by her father when engaging in conversations about social issues, unlike her dynamic with her mother:

They do [support my views]. Some of them, because I do have more open conversations with my mother because of things that have happened personally. But my father has always been, when it comes to political views and all that, he's always been standoffish about that. So we don't really have conversations about that.

Participants often had arguments with their parents about social issues, but some relationships were absent from that dialogue altogether. As in Valentina's case, this sometimes occurred with only one parent, so participants still had one pillar of support for dialogue.

Participants also described tensions with their parents' religious background, which influenced their conflicting sociopolitical values, typically related to sexist gender norms and homophobic messages at home. These differences in values made participants feel uncomfortable with discussing social issues with parents, which then limited further possible exploration of those social issues within and beyond the home. As an example, Fernanda (20, second‐generation Mexican) described how her parents' gendered beliefs made it difficult to socialize with peers because she was a girl. She detailed how this impacted her later socialization, saying,

Because, as a girl, I wasn't allowed to shake hands with guys and my mom would come and stalk me at my school after. I didn't have a boyfriend, but I would say bye to people and give a side hug and my mom's like, ‘Oh, that's your boyfriend.’ I would get accused and I was afraid to socialize after that.

Fernanda mentioned how she still struggles, not only with feeling comfortable to express herself in front of her parents, even when acknowledging that they had no bad intentions, but also that her parents' strictness stunted her ability to maintain friendships where positive conversations could occur. By enforcing these gender roles and limiting her interactions with peers on a gendered basis, her parents inadvertently limited her ability to discuss social issues with peers, and explore them further.

Cris (18, second‐generation Honduran) and a member of the LGBTQ+ community, describes how their parents' homophobic beliefs were judgmental and ultimately discouraged them from activism, which they reflected on by stating:

My parents, especially since they're homophobic, if I wanted to wear rainbow, they'd be like, ‘Oh, why are you wearing that? Are you this?’ They're kind of judgey about it. So I can't really be like, ‘Oh yes, pride!’ in front of them because they'd get suspicious. I'd tell my mom I support the community…my mom would make a face discouraging to speak out more.

Cris felt as though they could not demonstrate their support for their own community in front of parents because they would face discouragement or questioning of their own identity. Cris's experience with facing homophobia from parents was shared with other participants, who largely described how parental homophobia created differences between parent and child, which in turn created obstacles for mutual exploration and discussion of personally relevant social issues.

Additionally, participants noted that their parents worried about their child's safety while engaging in activism, particularly when attending protests. Other participants mentioned that they were fearful to engage in sociopolitical action alongside their parents who were undocumented, as they worried that taking a political stance might result in deportation or facing other obstacles. Sometimes parents' worries were more general, expressing concerns for their child's safety, especially at protests. Andrea (19, first‐generation Puerto Rican) discussed how her father expressed support for her attending protests outside of school but discouraged her participation in a school walk‐out. While her father encouraged her to focus on her studies, this message simultaneously discouraged her from using her voice within her community, prioritizing school over taking a stand. Ximena's mother more overtly voiced her disapproval of her attending protests in the community, specifically stating that she was concerned for her daughter's safety given the rioting involved in the 2020 protests. This discouragement eventually became too much for Ximena, who reflected,

Then at one point I didn't feel comfortable telling her that I was going to a protest because she'd just lecture me or she'd be like, ‘Oh, you shouldn't go. You should be very careful. You don't know. I don't like that.’ And I wouldn't say she stopped me, but like she wasn't very encouraging. I would say she was more scared of my safety even though there was no reason to be, but she just saw what was going on TV and she didn't want me to go.

Although Ximena still attended protests, her mother's discouragement created distance between them. Highlighting that while these tensions may create obstacles for youth sociopolitical development, parents were often coming from a place of love and care for their child. They wanted them to be safe, do well in school, and not place their lives or well‐being at risk.

#### Tension as a catalyst for sociopolitical development

For some participants, tensions with parents were instead a catalyst for their sociopolitical development. Allison (24, second‐generation Mexican) described this phenomenon, saying:

I guess they kind of dis‐influenced me to do some stuff, but at the switch of it, kind of motivates me to be like, no, like I'm gonna keep fighting for what I believe is right. And hope that my children and their children live way better than what my own parents went through, you know?

Allison also described how her conversations with her parents, even when they disagreed on social issues or argued, were motivating to continue her activism, “They definitely think differently than me, they're old school. If anything, they influence me to just keep doing what I'm doing now because I don't want future generations to be stuck where they're at.” The tensions created from varying opinions or interests was difficult for her relationship with her parents but provided motivation to change others' attitudes through her engagement.

Some participants also discussed how their parents simply seemed to be unaware of many social issues, or lacked the understanding of how those social issues impacted their own lives, which participants tried to explain to their parents. Parents' lack of knowledge was motivating for participants to explore independently and then engage with parents through teaching them about social issues. Nicolas (21, second‐generation Mexican) described how he experienced this with his parents, saying, “They wouldn't know most political issues, or they didn't see how it connected to them, we never talked about politics at home when I was growing up…So their lack of knowledge made me more interested in knowing.” His parents' lack of understanding, and subsequent lack of socialization toward social issues, spurred Nicolas' interest in learning more about them. While not overtly attempting to discourage his exploration, his parents' distance from social issues limited his early exposure to such discussions; yet this absence was motivating to Nicolas as a reason to explore.

Similarly, Mia explained how she saw her father's experiences as a Latino man altered his perspectives on social justice issues, which sometimes meant that he didn't quite see the alignment among parallel racial justice movements. She reflected on this, saying:

There's certain things [that are] a generational learning curve: mental health is something that they don't really get, especially my dad being a Hispanic man with a lot of machismo. Race, or what it's like growing up as a mixed person, or seeing a social issue like Black Lives Matter, they don't really get it entirely because they [think] everyone has it bad. My dad would tell me these stories where the police used to ask [him] to be in a lineup just because [he] match[ed] the description. I'm like, that's not okay, you're just conditioned to believe that that's okay and it's not. So I think that there's just a huge learning curve [between] their generation and mine.

Here, Mia described a common experience throughout the participants' reflections, which detailed how parents often believed that the treatment and oppression that they experienced was excusable simply because many people around them experienced it. Participants often described trying to teach their parents about systemic issues, or to understand that conditions in the US didn't have to be accepted without question just because conditions might have been worse in their countries of origin.

Further, having these conversations, even when in disagreement, helped participants' exploration of social issues because it allowed participants to gain other perspectives on the topic. Catalina described how she felt supported by her mom because her mom was engaging in dialogue about social issues with her, even when they disagreed on the issue, saying “I've had to talk to my mom a lot about this and we've come to the conclusion that she doesn't agree with abortion, but she has no right to enforce that opinion on other people.” Despite their differing views, her mom was able to compromise on dictating others' choice for abortions. Having these conversations, even when in disagreement, helped participants' exploration of social issues because it allowed participants to gain other perspectives on an issue.

## DISCUSSION

Our findings suggest that Latinx parents are engaging in myriad approaches to sociopolitical socialization. While the current study did not aim to study youth's sociopolitical values (i.e., personal priorities with implications for social and political issues; Bardi & Schwartz, 1996), it became clear that parental socialization practices were inextricably linked to youth developing their sociopolitical values. This finding might be due to the study's focus on adolescents as prior literature suggests that youth begin to develop their own sociopolitical values during this critical developmental period (Oosterhoff et al., 2017). Therefore, our findings further situate adolescence as a unique period opportune for the study of sociopolitical development. During this stage, youth are establishing their own identity distinct from their family unit while simultaneously receiving impactful messages from family members that influence their sociopolitical understandings. In our work, we found that this transmission of ideas primarily occurred through dialogue–critical conversations between parents and their children.

The conversations that youth recalled as being impactful for their sociopolitical values took two forms: direct and indirect socialization practices. While not all parents explicitly seek to orient their children toward sociopolitical issues, our participants recall poignant instances that indirectly informed the development of their own sociopolitical values. The indirect practices youth discussed highlighted how parents imparted both cultural and communal values, which produced a foundation that ultimately informed their own sociopolitical values in an indirect manner. Whereas direct socialization practices were more tangibly related to sociopolitical development, as these conversations were tied to specific aspects, like modeling and encouraging activist work. This is not to be confused with *overt* and *covert* forms of parental socialization (Hughes et al., 2023). It is important to note that within both the indirect and direct approaches we identified, parents engaged in both overt and covert socialization techniques. For instance, when imparting messages about their Latin American ancestry, participants recalled their parents highlighting their culture in covert or subliminal ways at home, like through food. At the same time, parents would overtly or explicitly discuss the importance of remembering one's heritage. These examples map neatly onto existing work showcasing how ethnic‐racial socialization can impart a strong ethnic‐racial identity onto Latinx youth (Vezaldenos et al., 2023; Umaña‐Taylor et al., 2014). Our findings enhance burgeoning scholarship suggesting that ethnic‐racial socialization at home also influences youth sociopolitical development (Rivas‐Drake et al., 2014). For example, youth that felt connected to their Latin American heritage described feeling an obligation to address issues that impacted their community. This finding builds off the limited quantitative literature exploring these associations and offers qualitative examples of what these practices look like within the homes of diverse Latinx immigrant‐origin youth.

Existing research on civic and political socialization supports the important role that parents play, particularly through sociopolitical discussions (Bañales, Aldana, et al., 2021; Bañales, Hope, et al., 2021; Diemer, 2012; McIntosh et al., 2007), but also through modeling civic behaviors, such as volunteering and showing concerns for others (White & Mistry, 2016; Wray‐Lake & Syvertsen, 2011). Similarly, our findings highlight how these sociopolitical socialization practices take place among Latinx families and expand the existing scholarship. In line with this past research, our findings suggest that for Latinx youth in our sample, family environments were particularly impactful when they explicitly encouraged sociopolitical engagement. This sometimes looked like parents themselves being engaged sociopolitical actors in their communities and bringing their children into the mix alongside them. Other times this looked like parents encouraging their children to become involved in issues that mattered to them and being supportive of that engagement. Unlike original studies on civic socialization, these practices identified in our study were inextricably linked to cultural values and identity as discussed above. Furthermore, our findings somewhat complicate existing work on political socialization, as the current study is the first to document the tensions that arise from such discussions at home.

Several of our participants noted that sociopolitical conversations with family sometimes resulted in tension or discomfort, often stemming from differences in sociopolitical views between parent and child. To our knowledge, this is the first empirical scholarship demonstrating how parents may pose *obstacles* to their child's sociopolitical development. This finding might be contextualized given our sample of primarily college‐aged youth. When navigating the transition to adulthood, college settings provide an optimal context for civic and social engagement, as they offer increased agency and autonomy (Bloemraad & Trost, 2008; Flanagan & Levine, 2010). This context may result in youth bringing up sociopolitical topics with their parents more frequently and being more steadfast in their stances on these issues. The resulting tensions demonstrate how, for late adolescents navigating a college context, youth that are attempting to exert more agency and autonomy regarding their sociopolitical beliefs may also produce tension at home with their parents.

These intergenerational tensions may also reflect broader acculturation dynamics within immigrant families. Researchers have thoroughly documented that immigrant‐origin adolescents live at the intersection of at least two cultures (Berry et al., 2006), and differing cultural values between youth and their parents—often referred to as an acculturation gap—may create intergenerational conflict (Birman, 2006). The tensions we observed around sociopolitical value may represent one manifestation of this gap, as youth who are more acculturated to the U.S. sociopolitical context may hold different perspectives than their parents on issues such as protest participation, political expression, or which social issues warrant attention.

Yet, it is important to note that not all tenuous conversations resulted in youth feeling limited in their sociopolitical engagement. Sometimes these interpersonal tensions served as a catalyst for youth to continue or deepen their engagement in social justice movements. Additionally, several participants noted that the tensions that became obstacles to their sociopolitical development were due to parental concerns regarding safety and success. This looked like parents expressing concern for their child's physical safety at protests, familial safety regarding risk for deportation, and encouraging children to instead focus on schooling and other contexts that will be beneficial for their child. While these messages may have discouraged youth from engaging deeper in social movements, parents in these cases were approaching such conversations with deep love and care for their child. The children often understood the emotions underlying their parents' sentiments and felt responsible for honoring their parents' concerns. This phenomenon aligns with existing work on familism within Latinx families (e.g., Gibbons, 2024; Steidel & Contreras, 2003), such that concerns for familial safety and well‐being were taken to heart by the youth in our study. This resulted in youth heeding their parents' concerns which sometimes resulted in lesser engagement in social issues.

### Limitations and future directions

The present study offers valuable insights into how Latinx emerging adults perceive their parents' role in shaping their sociopolitical development; however, several limitations should be considered when interpreting these findings. First, our reliance on retrospective accounts of parental socialization strategies introduces potential recall bias. Participants were asked to reflect on experiences that occurred years prior, which may have influenced the accuracy or completeness of their recollections. Future research would benefit from examining these processes during earlier developmental periods when socialization experiences are more proximal. High school may represent a particularly salient window, as several participants in our study identified this as a time of increased civic involvement. Additionally, because some youth described parents modeling sociopolitical engagement from an early age, researchers might consider longitudinal designs that capture these dynamics as they unfold across childhood and adolescence.

A related limitation concerns our single‐informant design. Although emerging adults provided rich descriptions of their parents' behaviors and attitudes, we did not capture parents' own perspectives on their socialization intentions or practices. Future studies seeking to understand the full scope of sociopolitical socialization would benefit from interviewing both youth and their parents. Parent–child dyads engaged in shared sociopolitical action—such as families who volunteer together or participate jointly in community organizing—may offer particularly fruitful contexts for examining how reciprocal influence operates in real time. Relatedly, the perspective of parents may help inform whether Latinx parents may play a role in the opportunity structures component of sociopolitical development theory. Watts and colleagues characterize opportunity structures as networks of organizations and resources that create an infrastructure for facilitating youth sociopolitical development, frequently involving non‐familial adults who partner with young people to pursue social change (Watts & Flanagan, 2007; Watts & Halkovic, 2022). The potential for family, particularly Latinx families, as a component of the opportunity structures is an emerging area of study (e.g., de los Reyes et al., 2025) that remains largely underexplored.

Similarly, future work might explore differences in sociopolitical socialization from Latinx mothers and fathers with their adolescent children. While the current study was designed to explore parental socialization broadly, our findings demonstrate extensive, albeit not exclusive, examples of sociopolitical socialization from participants' mothers. Some research suggests that mothers might play a unique role in the racial and civic socialization of their children (Atkin & Ahn, 2022; Balkaya‐Ince et al., 2020; Montgomery et al., 2024; Rollins & Hunter, 2013), but future research should more explicitly examine this relationship among immigrant‐origin Latinx youth.

Our findings also raise questions about the conditions under which familism supports versus constrains sociopolitical development that warrant further investigation. Although familism emerged as a meaningful influence on participants' civic trajectories, the mechanisms linking familism to sociopolitical outcomes remain unclear. Future quantitative research could explore whether this relationship is mediated by factors such as critical consciousness or ethnic‐racial socialization. Additionally, researchers might examine whether there is an optimal level of family loyalty for sociopolitical development—that is, whether very high levels of familism increase the likelihood that family members' concerns will inhibit or benefit youth sociopolitical development. Further, our inclusion criteria focused on Latinx youth who were interested in making a change in their community. Thus, we know less about the parental socialization practices of Latinx youth who are not interested in (or do not have the time) to engage in community change efforts. To better examine potential familial obstacles for youth sociopolitical development, future studies should include youth who are disengaged from any form of sociopolitical action.

While Latinx emerging adults represent an important population for studying sociopolitical development given their unique positioning at the intersection of immigration, ethnic‐racial identity, and civic life, our findings are specific to this context. Future researchers could examine whether similar socialization processes operate in other immigrant communities. For instance, do Black (non‐Latinx) immigrant families employ comparable modeling and encouragement strategies? Do tensions around sociopolitical involvement manifest similarly in Asian American immigrant families? How might these dynamics differ in multiracial households or between mothers and fathers? Comparative research across diverse populations would help clarify which aspects of sociopolitical socialization are culturally specific versus broadly shared. Finally, our results also have important implications for parent–child civics programs targeting Latinx families, suggesting the need for interventions that address both supportive and potentially restrictive parental practices.

## CONCLUSION

This study offers an in‐depth, qualitative examination of how immigrant‐origin Latinx youth perceive their parents' role in their sociopolitical development. Our findings show that parents shape youth sociopolitical development through both indirect socialization practices—affirming Latin American identity and imparting communal values—and direct socialization practices—modeling engagement and providing encouragement. Importantly, we also documented how intergenerational tensions, while sometimes hindering youth engagement, can also catalyze deeper commitment to social justice. These findings extend sociopolitical development theory through the development of a Latinx parental sociopolitical socialization theory, expanding the existing literature on early upbringing experiences. As Latinx youth continue navigating an increasingly contentious sociopolitical landscape, understanding the complex role of family relationships in fostering—and at times constraining—their civic trajectories remains essential for researchers, practitioners, and families alike.

## AUTHOR CONTRIBUTIONS

**Wendy de los Reyes:** Conceptualization; methodology; writing – original draft; investigation; funding acquisition; visualization; writing – review and editing; formal analysis; project administration; data curation; supervision; resources. **Victoria Vezaldenos:** Conceptualization; writing – original draft; visualization; writing – review and editing. **Corissa Draper:** Formal analysis; writing – review and editing; visualization; writing – original draft; conceptualization. **Rosaria Barraza:** Conceptualization; writing – original draft; visualization; writing – review and editing; formal analysis.

## FUNDING INFORMATION

This project received funding support from Nike (obtained by MENTOR: The National Mentoring Partnership), the DePaul University College of Science & Health, and the Irwin W. Steans Center for Community‐Based Service Learning & Community Service. The funding sources had no say in the study design, analysis of data, interpretation of results, or writing of the report.

## CONFLICT OF INTEREST STATEMENT

Authors state that there are no conflicts of interest to report.

## ETHICS STATEMENT

This study adhered to guidelines for the protection of human subjects and was approved by the DePaul University Institutional Review Board (#IRB‐2022‐596) on June 29, 2022.

## CONSENT STATEMENT

Informed consent was obtained from participants directly as they were all over 18 years old.

## Data Availability

The data that support the findings of this study are available on request from the corresponding author. The data are not publicly available due to privacy or ethical restrictions.
